# Functional characterization of four soybean C2H2 zinc-finger genes in *Phytophthora* resistance

**DOI:** 10.1080/15592324.2025.2481185

**Published:** 2025-03-20

**Authors:** Yuting Chen, Xinyue Liu, Yanyan Zhou, Yu Zheng, Yating Xiao, Xingxing Yuan, Qiang Yan, Xin Chen

**Affiliations:** a Institute of Industrial Crops, Jiangsu Academy of Agricultural Sciences/Jiangsu Key Laboratory for Horticultural Crop Genetic Improvement, Nanjing, China; b College of Life Sciences, Nanjing Agricultural University, Nanjing, China; c School of Life Sciences, Jiangsu University, Zhenjiang, China

**Keywords:** Soybean, *phytophthora* root rot, C2H2 zinc finger protein (C2H2-ZFP), GmZFP2, transcriptional repression

## Abstract

Soybean (*Glycine max*) is one of the most important industrial and oilseed crops; however, the yield is threatened by the invasion of various pathogens. Soybean stem and root rot, caused by *Phytophthora sojae*, is a destructive disease that significantly damages soybean production worldwide. C2H2 zinc finger protein (C2H2-ZFP) is a large transcription factor family in plants that plays crucial roles in stress response and hormone signal transduction. Given its importance, we analyzed the expression patterns of C2H2-ZFP family genes in response to *P. sojae* infection and selected four candidate genes to explore their molecular characteristics and functions related to *P. sojae* resistance. Subcellular localization analysis indicated that three ZFPs (GmZFP2, GmZFP3, and GmZFP4) were localized in the nucleus, while GmZFP1 was found in both the nucleus and plasma membrane. Dual-luciferase transient expression analysis revealed that all four ZFPs possessed transcriptional repression activation. Further transient expression in *N. benthamiana* leaves demonstrated that *GmZFP2* induced significant cell death and reactive oxygen species (ROS) accumulation. *GmZFP2* significantly enhanced the resistance to *Phytophthora* pathogens in *N. benthamiana* leaves and soybean hairy roots. This study provides insights in to the functional characterization of soybean ZFPs in *Phytophthora* resistance and demonstrates that *GmZFP2* plays a positive role in *P. sojae* resistance in soybeans.

## Introduction

In the long process of co-evolution between plants and pathogens, plants have developed a sophisticated immune system to defend against pathogenic infections. The plant immune system is currently recognized to be divided into two levels based on its resistance mechanisms: pathogen-associated molecular patterns (PAMP)-triggered immunity (PTI), which is activated by PAMPs, and effector-triggered immunity (ETI) activated through recognition of pathogen effector molecules.^1,2^ The activation of disease resistance signaling in plants is accompanied by genome-wide transcriptional reprogramming, such as nearly the entire genome genes (97–99%) of soybean undergo transcriptional modulation in response to *Phytophthora sojae* infection.^3^ In this process, transcription factors (TFs) play significant roles in the activation or repression of plant defense genes. TFs act as “molecular switches” turning on or off the transcription of their target genes through interaction with cis-elements located in the promoter region.^4^ Based on the primary sequences and 3D structures of binding domains, plant TFs have been classified into 40–60 families， including AREB, MYB, WRKY, bZIP, and ZFP.^5,6^

ZFP represents one of the largest TF families in the plant kingdom. They comprise one to several zinc fingers, typically range from 23 to 30 amino acids in length and have several cysteine (C) and/or histidine (H) residues.^7,8^ According to the number and location of C and H, ZFPs have been divided into 10 subclasses: C2H2, C2HC, C2HC5, C2C2, CCCH, C3HC4, C4, C4HC3, C6, and C8.^9^ Among these, C2H2-ZFP also named TFIIIA-type TFs, constitute the most abundant of ZFPs. In silico analysis of *Arabidopsis thaliana* reveals approximately 0.7% of encoded proteins possess C2H2 domains.^10^ All C2H2-ZFPs contain the conserved sequence C-X2∼4-C-X3-P-X5-L-X2-H-X3-H (X represents any amino acid), which is composed of approximately 30 amino acids. In plants, the number of C2H2 zinc fingers can range from one to dozens, providing the flexible affinity of DNA binding and suggesting their extensive biological functions.^11,12^

To date, C2H2-ZFPs have been systematically analyzed at the genome-wide level in many crops, such as rice,^13^ wheat,^14^ cucumber,^15^ potato,^16^ tomato,^17^ cotton,^18^ soybean,^19^ winter rape,^20^ sorghum,^21^ *Medicago truncatula*,^22^ ginseng,^23^ grapevine,^24^ and citrus.^25^ Emerging evidence indicates their pleiotropic roles in plant growth, development, hormone signal transduction, and stress resistance. Among the model plant *A. thaliana*, the ZFP TFs have been extensively characterized and are relatively well understood. ZFP5 plays a crucial role in trichome initiation by directly targeting ZFP8 expression through gibberellin (GA) signaling.^26,27^ Later research demonstrated that ZFP5 showed multifunction on root hairy elongation through ethylene signaling.^28^ Three root cap outermost cell-specific ZATs (ZAT1, ZAT4, and ZAT9) suppress the growth of *Arabidopsis* and regulate the maturation of the cells.^29^ Notably, AtZAT10 showed dual roles, as both overexpression and RNAi lines exhibit enhanced osmotic stress, salinity, and heat stress.^30^ AtZAT12 has been reported to be involved in cold and oxidative stress tolerance.^31^

Several C2H2-ZFPs of soybean have been isolated and were identified to play crucial roles in abiotic stress tolerance. Transgenic *A. thaliana* plants overexpressing soybean *SCOF-1* exhibited significantly enhanced freezing tolerance, further observations suggesting that SCOF-1 functions as a positive regulator of *COR* gene expression via interaction with SGBF-1.^32^ The expression of *GmZF1* responds to low temperature and exogenous ABA, and overexpression of *GmZF1* in *Arabidopsis* confers enhanced cold stress tolerance by regulating the expression of cold-responsive genes.^33^ In contrast, *GmZFP3* has been shown to negatively regulate drought response in an ABA-dependent pathway characterized in transgenic *Arabidopsis*.^34^ While *GmZAT4* contributes to Polyethylene Glycol (PEG) and NaCl stress tolerance.^35^ In addition, *GmZFP7* has been implicated in isoflavone biosynthesis through regulating the expression of *GmIFS2* and *GmF3H1*, and isoflavones are recognized for their critical roles in stress tolerance.^36^ These results demonstrate the functional diversity of C2H2-ZFPs in abiotic stress tolerance.

C2H2-ZFPs have also been shown to play important roles in plant disease resistance. In *Capsicum annuum*, the zinc finger protein CaPIF1 has been shown to confer enhanced bacterial resistance through constitutive upregulation of multiple defense-related genes.^37^ A single nucleotide change (A to G) in the promoter of rice *bsr-d1*, which encodes a C2H2 transcription factor, confers broad-spectrum resistance to rice blast.^38^ Recently, *GmZFP03* was identified as a resistant gene against *Phytophthora sojae* PsMC1 in the resistant soybean cultivar Yudou 29 through map-based strategy. *GmZFP03* specifically binding to a new DSREL motif and activating two *SOD1* genes.^39^

Soybean root and stem rot caused by *P. sojae* is a devastating disease, resulting in annual yield losses of $1–2 billion globally.^40^ Elucidating the molecular mechanisms underlying soybean and *P. sojae* interaction is essential for understanding pathogenesis and developing disease control strategies. In this study, by using transcriptome data before and after inoculation of two soybean varieties with different resistance levels to *P. sojae*, we analyzed the expression patterns of C2H2-ZFP family genes in response to *P. sojae* infection, and four candidate genes were selected for further functional detection. We first performed subcellular localization studies, followed by transactivation and/or transcriptional repression activations using the Y2H and dual-luciferase transient expression assay. Furthermore, the possible roles in triggering plant immunity and *P. capsici* resistance were investigated through transient overexpression assays in *N. benthamiana* leaves. Finally, we confirmed the resistant function of the selected ZFPs against *P. sojae* in transgenic soybean hairy roots. This study provides insights for the functional characterization of soybean ZFPs in pathogen resistance.

## Materials and methods

### Plant and pathogen cultivation

The cultivated soybean (Williams) and *N. benthamiana* seeds were sown in styrofoam pots containing a mixed substrate (peat: vermiculite: perlite = 2:1:1) and maintained in a greenhouse at 25°C, under 16 h:8 h light/dark photoperiod. The *Phytophthora* pathogens *P. capsici* isolate LT263, *P. sojae* isolate P6497 were cultured on 10% V8 medium and incubated in dark at 25°C.

### Gene cloning and plasmid construction

The full-length cDNA of *GmZFP1* (741 bp), *GmZFP2* (993 bp), *GmZFP3* (801 bp), and *GmZFP4* (1377 bp) were amplified by PCR and then cloned into pBIN-GFP4 vector between *Kpn*1 and *Bam*HI. In the recombinant plasmids, the ZFP genes were fused to the N-terminal of GFP and driven by CaMV35S promoter. The plasmids were transferred into *Agrobacterium tumefaciens* strain GV3101 using a freeze–thaw method for subcellular localization assays and transient expression in *N. benthamiana* leaves. The plasmids were transferred into *A. rhizogenes* strain K599 used for soybean hairy root transformation. All the primers used in this study are listed in Table S1. The sequence data of the four ZFPs have been submitted to the GenBank databases (GenBank: PQ761025-PQ761028).

### Subcellular localization assay

*Agrobacterium tumefaciens* GV3101 strain harboring the constructs was cultured in liquid LB supplemented with 50 mg/mL kanamycin. The culture was then washed and resuspended in infiltration medium (10 mm 2-Morpholinoethanesulphonic acid (MES), 10 mm MgCl_2_, 150 µM acetosyringone, pH 6.0) to make an appropriate optical density (OD) of 0.4 to 0.5 at 600 nm. Cultures harboring respective constructs, along with the DsRed-fused nucleus-localized signal (NLS), were co-infiltrated into four-week-old *N. benthamiana* leaves in a 1:1 ratio using a blunt syringe. After incubating in the growth chamber for 48 h, the infiltration regions were visualized with a laser scanning confocal microscope (Zeiss, Germany). The green fluorescent protein (GFP) and red fluorescent protein (RFP) fluorescence was excited at 488 and 561 nm, respectively.

### Transactivation assay in yeast

To assess the transactivation activity of GmZFP1, GmZFP2, GmZFP3, and GmZFP4, the CDS sequences of these genes were cloned into pGBKT7 vector between *Eco*RI and *Bam*HI. The constructs of pGBKT7-GmZFP1 ~4 + pGADT7-EV (empty vector), pGBKT7–53 + pGADT7-RecT (positive control), and pGBKT7-Lam + pGADT7-RecT (negative control) were co-transformed into yeast strain AH109 chemically competent cells (Coolaber, China). Transformants were cultured on SD/-Trp, SD/-Trp/-His/-Ade and SD/-Trp/-His/-Ade/+X-α-Gal medium plates for 3–4 d at 30°C.

### Dual-luciferase transient expression assay in Nicotiana benthamiana leaves

The sequences of Ω-GAL4 DBD-MCS1 (*Xba*I, *Spe*I, *Bam*HI)-VP16-MCS2 (*Eco*RI, *Hind*III, *Cla*I, *Kpn*I) and Ω-GAL4 DBD were synthesized (Tsingke, China), and subsequently cloned into the pGreenII62 plasmid between *Sac*I and *Kpn*I. The two vectors were designated as GAL4-DBD and GAL4-DBD-VP16, respectively. GAL4-DBD served as the negative control, while GAL4-DBD-VP16 acted as the positive control. The 5×GAL4-TATA sequence was synthesized and cloned into the pGreenII0800 plasmid between *Kpn*I and *Nco*I, which served as the reporter. The Renilla luciferase gene driven by the 35S promoter in pGreenII0800 was used as the internal control. In the transactivation assay, the CDS sequences of *GmZFP1*~*4* were cloned into GAL4-DBD-VP16 between *Bam*HI and *Kpn*I digestion sites. In the transcription repression test, the CDS sequences were incorporated into GAL4-DBD-VP16 via *Bam*HI restriction site.

Recombinant plasmids were transformed into GV3101 (pSoup-p19) chemical competent cells. Subsequently, the bacterial cultures harboring effector and reporter were co-infiltrated into *N. benthamiana* leaves. After incubation for 48 h, LUC/REN activity assays were performed with the dual-luciferase Reporter Assay System (Promega, USA) and photographed using the Tanon 5200 Multi Chemiluminescent Imaging System with an exposure time of 10 min.

### Transient expression and physiological assay

After agroinfiltration, the plants were maintained under normal growth conditions, and phenotypic observations were recorded at 1, 3, 5, and 7 days post agroinfiltration (dpa). To quantify the degree of cell death, electrolyte leakage was measured. Five leaf discs (5 mm in diameter) were excised from the agroinfiltration region and immersed in deionized water for 30 min at 25°C. Conductivity was measured using a FE32-Meter conductivity meter (ETTLER TOLEDO, Switzerland) and recorded as value “A”. After boiling in sealed tubes for 5 min, the samples were cooled to room temperature, and ion conductivity was measured again to obtain value “B”. Relative electrolyte leakage was calculated as (value A/value B) × 100. All assays were performed with three biological replicates. Accumulation of hydrogen peroxide (H_2_O_2_) was assessed by staining with 3,3’-diaminobenzidine (DAB). The infiltrated leaves were immersed in 1 mg/mL DAB (pH 4.0) solution and placed in the dark overnight at 37°C. Subsequently, the leaves were bleached in ethanol and visualized under white light.

### Soybean hairy root transformation

*A. rhizogenes* strain K599 harboring the constructs was cultured in liquid LB with 50 mg/mL kanamycin at 200 rpm for 24 h at 28°C. Following this, 0.1 mL of the *Agrobacterium* culture was spread onto LB plates (supplemented with 50 mg/mL kanamycin) and incubated 24 h at 28°C. The bacteria from the plates were then collected with a spreader and used for soybean infection.

Healthy soybean seedlings with unfolded green cotyledons were selected, and the cotyledonary node was wound with a scalpel. Approximately 5–10 μL of bacterial inoculum was applied to the wound. The treated plants were then placed in the greenhouse and covered with a transparent polyvinyl chloride cover. When hairy roots grew from the wounded sites, the primary roots were removed by cutting the stem 1 cm below the hairy roots. Positive hairy roots were detected by LUYOR-3415RG blue light flashlight (Luyor, China). After this, the plants were transferred to one-half Hoagland’s solution.

### DNA, RNA isolation, and quantitative RT-PCR

Following the supplier’s recommended protocol, genomic DNA and RNA samples were extracted using the Hi-DNA secure plant kit and the RNA simple Total RNA kit (Tiangen, China), respectively. Subsequently, cDNA was synthesized using the HiScript II Q RT SuperMix (+gDNA wiper) reagent kit (Vazyme, China). The qRT-PCR reactions were performed on a Roche LightCyclerr 480II instrument using the AceQ Universal SYBR qRT-PCR Master Mix (Vazyme, China). The relative gene expression levels were calculated using the comparative 2^−△△CT^ method.

### Pathogen infection assays

To evaluate the resistance of agroinfiltrated *N. benthamiana* leaves, *P. capsici* mycelium plugs were inoculated 24 h post-agroinfiltration (hpa). Lesion diameters were measured at 36 and 48 h post-inoculation (hpi). For soybean hairy root inoculation, *P. sojae* mycelia agar (~3 mm^3^) was inoculated at the elongation zone of positive roots. The resistant levels were quantitated by measurement of the relative biomass of *P. sojae* in infected hairy roots using qRT-PCR as above described. The primers PsTEF-F/R and GmCons4-F/R were used as internal references for *P. sojae* and soybean, respectively.^41^ Three independent biological replicates were conducted. The phenotypes of inoculated hairy roots were visualized under a macroview fluorescence microscope (OLYMPUS MVX10, Japan) at 48 hpi.

## Results

### *Expression analysis of soybean C2H2-ZFPs response to* P. sojae *infection*

**Figure 1.**
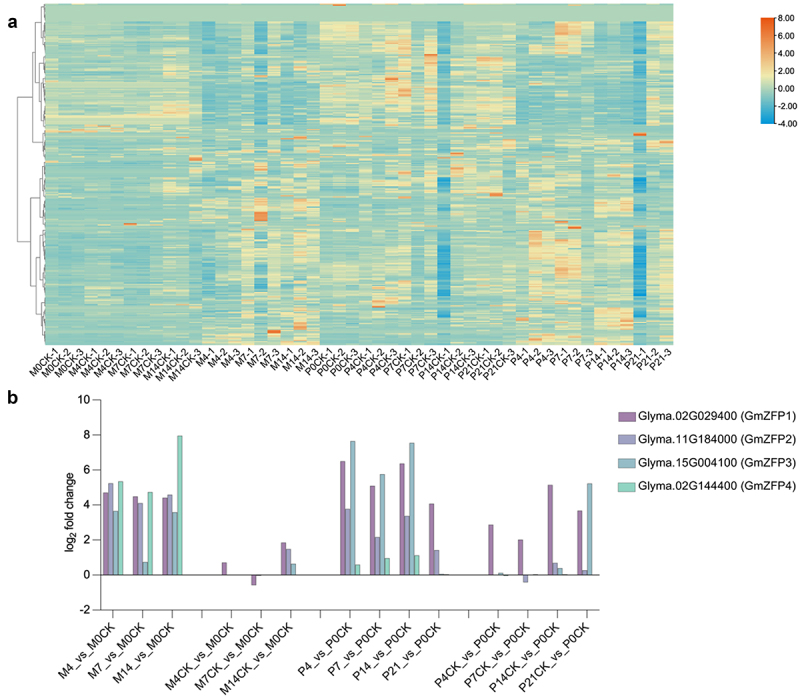
Expression of soybean C2H2 ZFP genes in response to *P. sojae* infection. (a) Expression profiles of soybean C2H2 ZFP genes in resistant (PI449459) and susceptible (misty) varieties 0, 4, 7, 14, and 21 d after *P. sojae* infection or mock-infection (CK). Heat maps were expressed using log_2_ values of transcriptome data. The color scale represents the relative expression levels from low (blue) to high (orange). (b) The log_2_ fold changes of *GmZFP1*, *GmZFP2*, *GmZFP3*, and *GmZFP4* response to *P. sojae* infection. The log_2_ values of gene expression changes were calculated for 4, 7, 14, and 21 d after *P. sojae* infection or mock-infection relative to that of 0 d. M: Misty; P: PI449459; CK: mock-infection; the numbers 0, 4, 7, 14, and 21 mean the days after infection.

To explore the potential roles of soybean C2H2-ZFPs in *P. sojae* resistance, a heatmap of 321 soybean C2H2-ZFP genes was established by using the transcriptome data obtained from NCBI (https://www.ncbi.nlm.nih.gov/bioproject/PRJNA574764). PI449459 exhibits high resistance to *P. sojae*, while Misty is highly susceptible.^42^ The expression patterns were classified into two clearly distinguishable subsets: Class I displayed a downregulation trend in both resistance and susceptible varieties compared to the non-inoculated controls (CK), while Class II exhibited upregulation in both varieties after inoculation (Figure 1a). The results indicated that the expression of soybean C2H2-ZFPs is significantly responsive to *P. sojae* infection. Based on the differential expression analysis, four candidate genes were selected (Table S2). *Glyma.02G029400* (*GmZFP1*) and *Glyma.11G184000* (*GmZFP2*) exhibited the highest levels of upregulation in both varieties compared to their respective 0 d CK. *Glyma.15G004100* (*GmZFP3*) showed markedly greater upregulation in the resistant variety, while *Glyma.02G144400* (*GmZFP4*) was specifically upregulated in the susceptible variety (Figure 1b). The four candidates of soybean C2H2-ZFPs have been used for further investigation.

### Subcellular localization of GmZFP1 ~ GmZFP4

**Figure 2.**
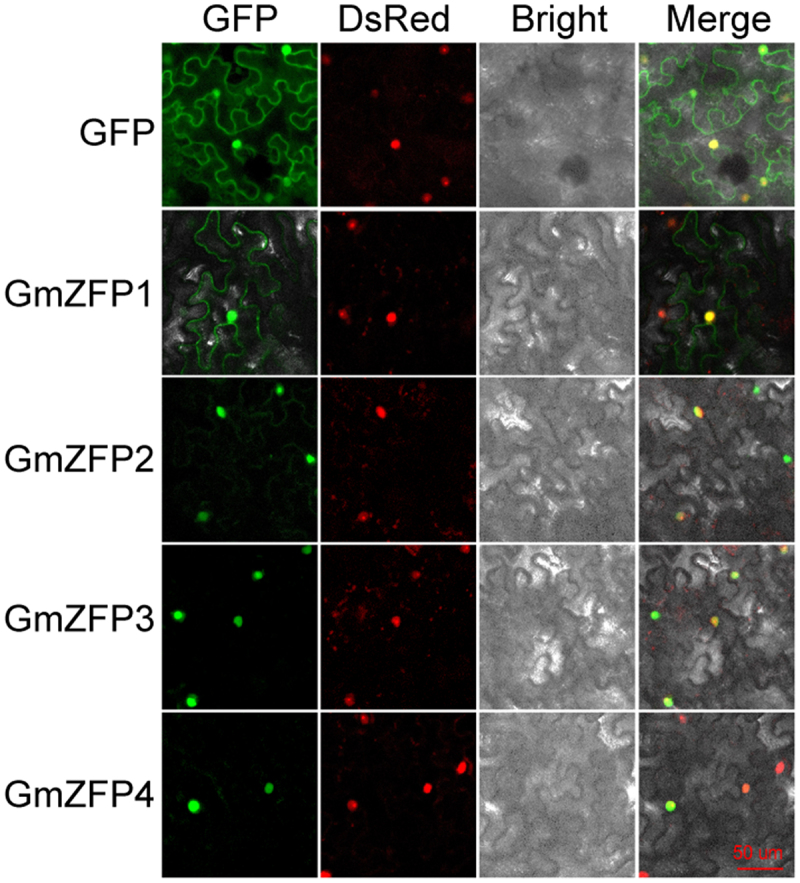
Subcellular localization of GmZFP1, GmZFP2, GmZFP3, and GmZFP4 in *N. benthamiana* leaf cells. Green and red fluorescence represent the signal of GFP or ZFP-GFP fusion protein and nuclear marker NLS-DsRed, respectively. Panels show in monochrome GFP, DsRed, bright field, and overlayed images for co-localization analysis. Bar = 50 μm.

To examine the subcellular localization of the four C2H2-ZFP candidates, the coding sequences of *GmZFP1* ~ *GmZFP4* were fused in-frame to the 5’ end of GFP. The fluorescence detection showed that GmZFP2, GmZFP3, GmZFP4, and the nucleus-localized signal (NLS) fused to DsRed were exclusively localized to the nucleus of the *N. benthamiana* leaf cells. GmZFP1-GFP displayed a distinct localization pattern, being found not only in the nucleus but also on the plasma membrane (Figure 2). Analysis of the protein structure and characteristics of GmZFP1 showed that GmZFP1 had nuclear localization signal – KKERMKKKR at positions 101–109, a small hydrophobic region at the C-terminus, and multiple phosphorylation sites (Supporting information 1). The results imply that GmZFP1 may be a membrane-bound transcription factor.

### GmZFP1 ~ GmZFP4 possess transcriptional repression activation

**Figure 3.**
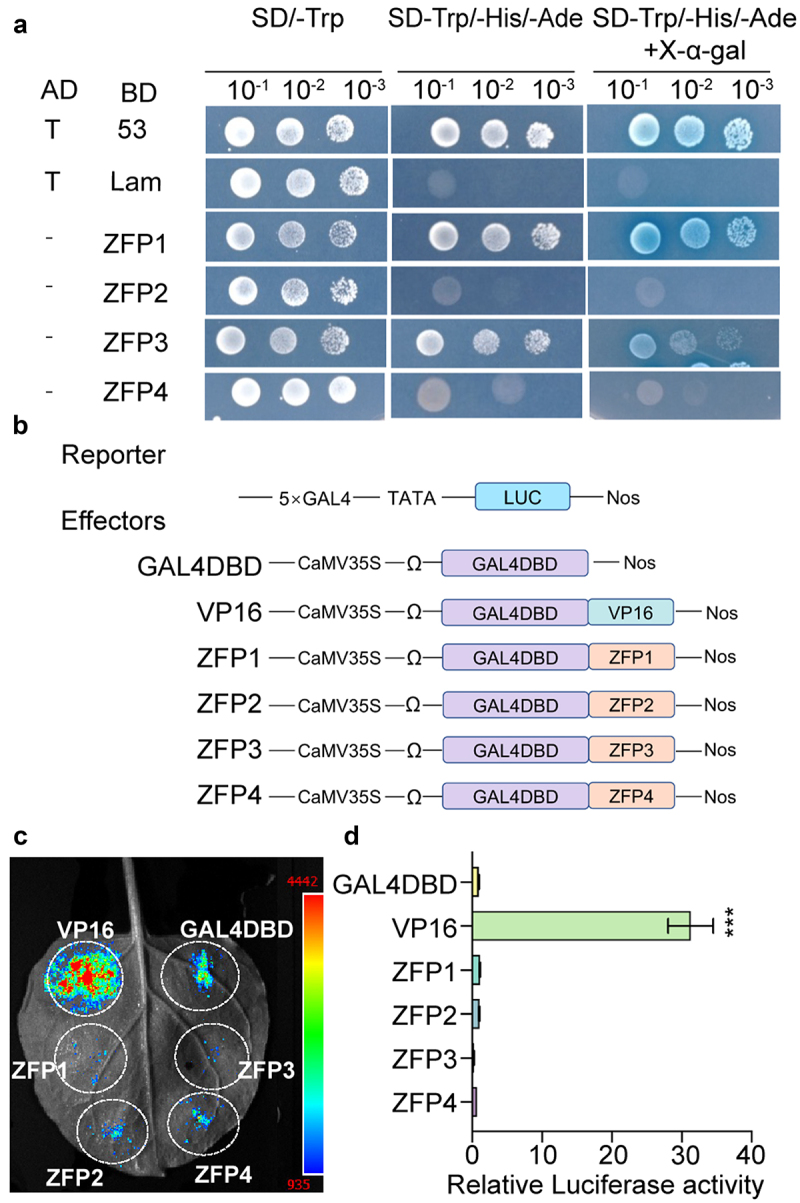
Transcriptional activation analysis of four soybean ZFP proteins. (a) Transcriptional activation analysis in yeast cells. pGADT7-T + pGBKT7–53 and pGADT7-T + pGBKT7-lam were used as positive and negative controls, respectively. (b) Schematic diagrams of the effector and reporter constructs used for transcriptional activation analysis in *N. benthamiana* leaves. (c) Transcriptional activation was analyzed in *N. benthamiana* leaves. The *Agrobacterium* harboring the GAL4-LUC reporter and GAL4 fusion vectors were co-infiltrated into the *N. benthamiana* leaves. Luciferase activity was detected at 48 hpa and photographed. (d) Quantitative analysis of transcriptional activation in *N. benthamiana* leaves with LUC/REN activity. Relative luciferase activity was measured at 48 hpa. GAL4DBD and GAL4DBD-VP16 were used as negative and positive controls, respectively. Asterisks in D indicate significant differences compared to GALDBD determined by one-way analysis of variance (ANOVA) (***p < 0.001). Data are mean ± standard deviation (SD) (*n* = 3).

To evaluate the functionality of the four C2H2-ZFPs as transcription factors, the transactivation abilities of GmZFP1, GmZFP2, GmZFP3, and GmZFP4 were analyzed in both yeast cells and *N. benthamiana* leaf cells. The CDS of the four genes were fused to the GAL4 DNA-binding domain (GAL4-DBD), respectively. As shown in Figure 3a, all the transformants grew well on the SD-Trp plates, only GmZFP1 exhibited strong transactivation abilities, and GmZFP3 weakly activated the transcription of the GAL4 reporter gene, while GmZFP2 and GmZFP4 showed no transactivation abilities. We further examined the transactivation using a dual-luciferase reporter assay in *N. benthamiana* leaves. The ZFP genes fused to GAL4-DBD served as effectors, and the luciferase gene driven by CaMV35S with five copies of the GAL4 binding element was used as reporter (Figure 3b). After transient co-expression in *N. benthamiana* leaves, the luciferase activity of the four ZFP genes was all significantly lower compared to the positive control BD-VP16 and showed no significant difference to GAL4-DBD (Figure 3c,d). Collectively, these results suggest that the four ZFP genes either lack transcriptional activation activity or may function as transcription repressors in plants.

**Figure 4.**
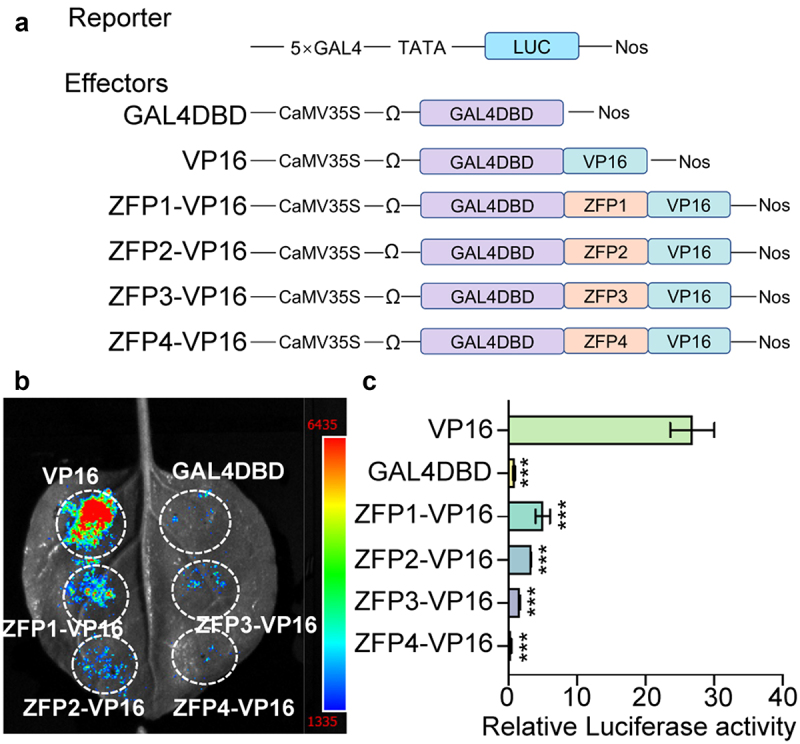
Effect of GmZFP1, GmZFP2, GmZFP3, and GmZFP4 on VP16 activity in *N. benthamiana* leaves. (a) Schematic diagrams of the effector and reporter constructs used for transcriptional repression activation analysis in *N. benthamiana* leaves. GAL4DBD and GAL4DBD-VP16 were used as negative and positive controls, respectively. Soybean ZFP was fused to the N-terminal of VP16 to test its effects. (b) Luciferase fluorescence was observed in *N. benthamiana* leaves at 48 hpa. (c) Quantification of LUC/REN activity in transcriptional repression activation analysis. The reporter and effector were co-infiltrated into *N. benthamiana* leaves and relative luciferase activity was measured at 48 hpa. Asterisks in C indicate significant differences compared to VP16 determined by one-way ANOVA (***p < 0.001). Data are mean ± SD (*n* = 3).

To confirm the repressive function of GmZFP1, GmZFP2, GmZFP3, and GmZFP4, fusion proteins of ZFP with VP16 were used as effectors (Figure 4a). The results of dual-luciferase reporter assay showed that the luciferase activities of the four ZFP genes were significantly reduced compared to BD-VP16 (Figure 4b,c). The fusing of ZFP with VP16 inhibited 81% (GmZFP1) to 99% (GmZFP4) of the original VP16 activity (Figure 4c). These results indicate that the four ZFP genes may function as transcription repressors.

### GmZFP2 induces ROS accumulation and cell death

**Figure 5.**
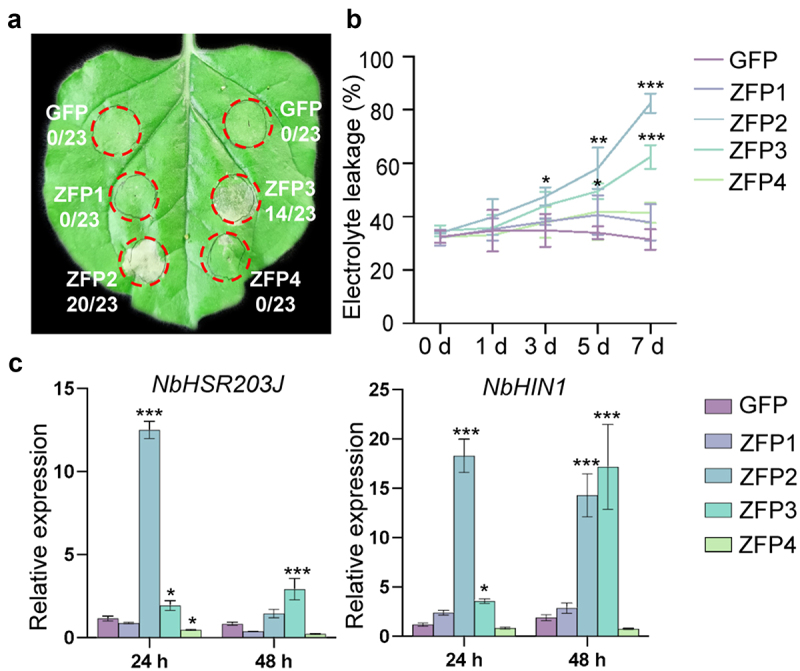
The cell death induction activity detected in *N. benthamiana*. (a) Transient expression of *GmZFP1*, *GmZFP2*, *GmZFP3*, and *GmZFP4* in *N. benthamiana* leaves by agroinfiltration. The ratios represent the number of cell death to the total number of infiltration sites. Photographs were taken at 5 dpa. (b) Quantifications of electrolyte leakage at 1, 3, 5, and 7 dpa. (c) Relative expression of two marker genes related to hypersensitivity response (HR) in *N. benthamiana* leaves. Agroinfiltrated leaves were sampled at 24 and 48 hpa to detect the relative expression of *NbHIN1* and *NbHSR203J* by qRT-PCR. Transcript levels of genes were normalized to the reference gene *NbEF1a*. GFP was used as a negative control, and the values of GFP were normalized as 1. Asterisks in B and C indicate significant differences determined by one-way ANOVA compared to GFP control (***p < 0.05; ***p < 0.01; *p < 0.001). Data are mean ± SD (*n* = 3).

After 5 d of transient expression of *GmZFP2* and *GmZFP3* in *N. benthamiana* leaves, obvious cell death symptoms were observed in agro-infiltration regions, *GmZFP2* induced cell death more rapidly than *GmZFP3* (Figure 5a). Cell death was noticeably evident 2 dpa for *GmZFP2*, while it was detectable 3 dpa for *GmZFP3*. Furthermore, *GmZFP2* exhibited a stronger induction of cell death compared to *GmZFP3*. The electrolyte leakages of *GmZFP2* and *GmZFP3* overexpression leaf discs were significantly elevated since 3 dpa, and the conductivity of *GmZFP2* was significantly higher than that of *GmZFP3* at 7 dpa (Figure 5b). Additionally, we further determined the expression of two marker genes related to hypersensitive response (HR) in *N. benthamiana*. The results showed that *GmZFP2* significantly activated the expression of *NbHSR203J* at 24 hpa and *NbHIN1* at both 24 and 48 hpa. In contrast, *GmZFP3* showed weaker induction of *NbHSR203J* compared to *GmZFP2*, but still significantly higher than *GFP*, *GmZFP1*, and *GmZFP4*. The expression of *NbHIN1* was significantly elevated at 48 hpa only (Figure 5c).

**Figure 6.**
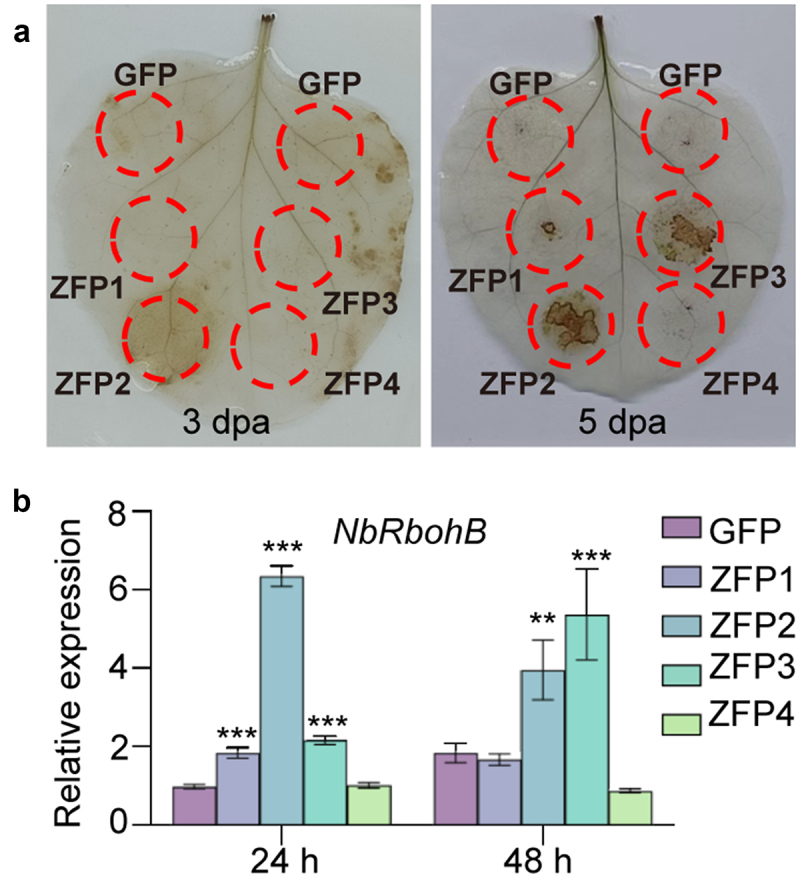
H_2_O_2_ induction activity detected in *N. benthamiana*. (a) H_2_O_2_ accumulation detected by DAB staining in *N. benthamiana* leaves at 3 and 5 dpa. (b) The expression of *NbRbohB* in *N. benthamiana* leaves at 24 and 48 hpa. *NbEf1a* was used as an internal reference gene, and the value of GFP was normalized as 1. Asterisks in B indicate significant differences determined by one-way ANOVA compared to GFP control (***p < 0.01; ***p < 0.001.). Data are mean ± SD (*n* = 3).

ROS burst is usually important for the initiation of HR. Hence, we performed DAB staining at 3 and 5 dpa. The results showed that *GmZFP2* significantly induced ROS accumulation at 3dpa, and pronounced ROS accumulation observed in both *GmZFP2* and *GmZFP3* overexpression regions at 5 dpa (Figure 6a). The expression of respiratory burst oxidase homolog *NbRbohB* was markedly up-regulated in *GmZFP2*-expressing leaves at both 24 and 48 hpa while only elevated at 48 hpa in *GmZFP3*-expressing leaves (Figure 6b). These results indicate that *GmZFP2* possesses a significant capacity to induce cell death in *N. benthamiana*, accompanied by ROS burst.

### *GmZFP2 enhances resistance to* Phytophthora *in plants*

**Figure 7.**
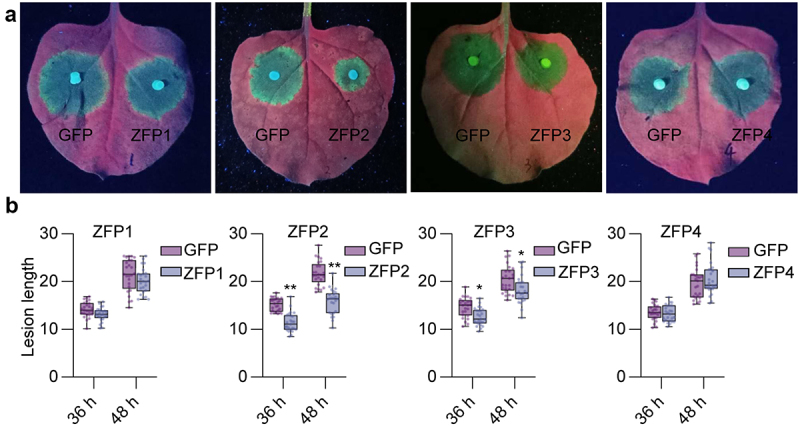
Resistant evaluation of *GmZFP1*, *GmZFP2*, *GmZFP3*, and *GmZFP4* transiently overexpressing in *N. benthamiana* leaves to *P. capsici*. (a) Phenotypes of lesions on *N.Benthamiana* leaves at 48 hpi with *P. capsici*. Representative photographs were taken under ultraviolet (UV) light. (b) The measurements of the average lesion length at 36 and 48 hpi. Asterisks indicate significant differences determined by the Student’s *t*-test (***p < 0.05; ***p < 0.01). Data are mean ± SD (*n* = 25).

As HR and ROS accumulation are common features of plant immune responses, we investigated the involvement of the four ZFP genes in *Phytophthora* resistance. We initially expressed GFP and *GmZFP1* ~ *4* in *N. benthamiana* leaves by using *A. tumefaciens*-mediated transient expression. The leaves were inoculated with *P. capsici* mycelia plugs at 24 hpa; overexpressing *GmZFP2* significantly reduced lesion diameter at both 36 and 48 hpi, overexpressing *GmZFP*3 also showed enhanced resistance but was weaker than *GmZFP2* (Figure 7a,b). The results imply that *GmZFP2* significantly enhanced the resistance to *P. capsici* in *N. benthamiana*.

**Figure 8.**
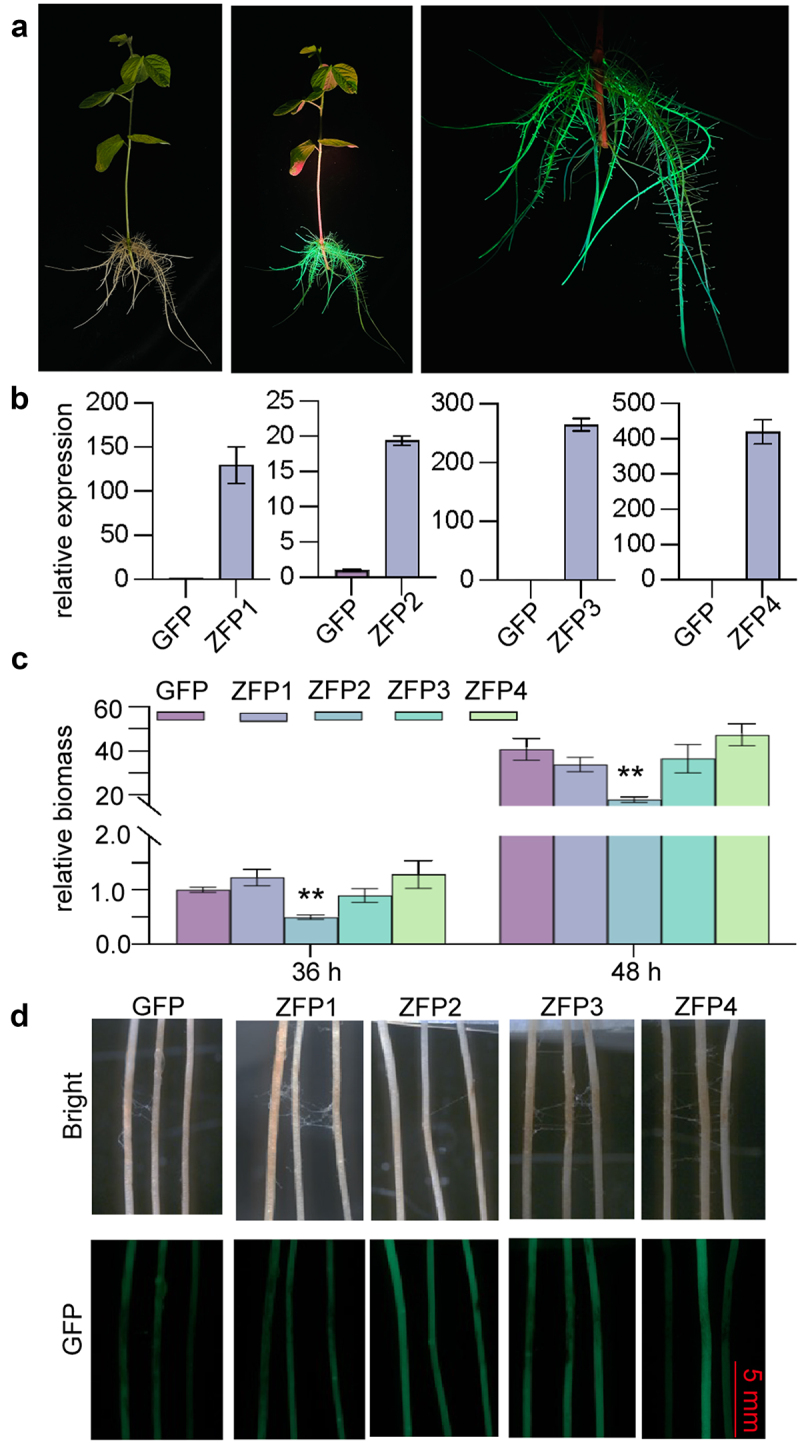
Resistant evaluation of *GmZFP1*, *GmZFP2*, *GmZFP3*, and *GmZFP4* overexpressing soybean hairy roots to *P. sojae*. (a) GFP fluorescence screening of transgenic soybean hairy roots. The transformed chimeric plants were photographed under bright and blue light, respectively. (b) Relative expression levels of indicated ZFPs in transgenic soybean hairy roots. The expression levels of respective ZFPs in GFP transgenic soybean hairy roots were normalized as 1. *GmCons4* were used for internal reference. (c) Relative biomass accumulation of *P. sojae* in the inoculated soybean hairy roots. *GmCons4* and *PsTEF* were used for relative biomass detection by qRT-PCR. The relative biomass at 36 hpi in GFP transgenic hairy roots was normalized as 1. (d) Phenotypes of soybean hairy roots at the infection site. Photographs were taken at 48 hpi using a macroview fluorescence microscope with bright and fluorescence, respectively. Mycelia plugs were removed from the roots before photography. Asterisks in C indicate significant differences determined by one-way ANOVA compared to GFP control (***p < 0.01). Data are mean ± SD (*n* = 3).

By using *A. rhizogenes*-mediated hairy root transformation, we obtained transgenic soybean hairy roots overexpressing GFP and *GmZFP1* ~*4* respectively. The positive hairy roots were initially screened through green fluorescence observation (Figure 8a). The expression of target gene in transgenic hairy roots was examined by qRT-PCR, the expression levels of *GmZFP1*, *GmZFP3*, and *GmZFP4* were over 100-fold higher than those of GFP control (Figure 8b). The expression level of *GmZFP2* increased approximately 20-fold, which may be attributed to the cell death-inducing effect (Figure 8b). Nevertheless, we successfully obtained the *GmZFP2* overexpressing hairy roots. After inoculation with *P. sojae*, the relative biomass in *GmZFP2* overexpressing hairy roots was significantly reduced at both 36 and 48 hpi (Figure 8c). At 48 hpi, the inoculation zone of the hairy roots displayed pronounced brown lesions, with hyphae noticeably extending across the roots surface. The fluorescence of the hairy roots gradually diminished and eventually disappeared due to tissue necrosis. The hairy roots overexpressing *GmZFP2* exhibited significantly less severe disease symptoms compared to control and those overexpressing the other three ZFP genes, the infected roots still retained strong green fluorescence (Figure 8d). Taken together, the results demonstrated that *GmZFP2* enhances resistance to *Phytophthora* pathogens.

## Discussion

Modern plants possess a highly complex immune repertoire with diverse signal perceptions and intracellular signaling pathways. Transcription factors (TFs) frequently serve as convergence points for signals, and efficient signal transduction results in the activation of TFs, thereby regulating the processes involved in plant immunity.^43^ Numerous transcription factors have been identified as mediators of soybean resistance to *P. sojae*. In this study, we evaluated the function of four ZFP genes in soybean resistance to *P. sojae*, and demonstrated that *GmZFP2* plays an important role in *P. sojae* resistance. From the results, we can speculate that C2H2 ZFP transcription factors are also largely involved in soybean and *P. sojae* interaction.

With the increasing publishing of plant genomes and the extensive investigation of C2H2 ZFP genes at the genome-wide level across various plants, the comprehension of the C2H2 zinc finger proteins is also deepening. C2H2 ZFPs typically possess the plant-specific “QALGGH” motif within the ZFP domain. The “QALGGH” motif is critical for the DNA-binding activity.^44^ The other main motif that embodies this family is the C-terminal EAR (ERF-associated amphiphilic repression) motif, which serves as an active repressor domain.^45^ In soybeans, the 321 C2H2 ZFPs are categorized into 11 subfamilies, with only the Q class possessing the conserved QALGGH motif. In this study, GmZFP1 and GmZFP3 are classified as typical Q subfamily members, possessing an invariant QALGGH motif, whereas members GmZFP2 and GmZFP4 lack the QALGGH motif. Wheat TaZF possesses typical “QALGGH” and EAR motifs. The mutants with amino acid substitutions or deletions were shown to have a reduced cell death response compared with the wild-type, but the interaction with AvrPm2 is not influenced.^46^ The results reveal that the QALGGH motif plays an important role in C2H2 ZFP genes. However, GsZFP1 lacking the typical QALGGH motif acts as a positive regulator to enhance cold and drought tolerance and negatively regulates ABA signaling in *A. thaliana*.^47,48^ In our study, GmZFP2 also lacks the QALGGH motif, suggesting that in certain subsets of C2H2 ZFP genes that lack QALGGH motif, other critical motifs may be present that are essential for their function.

Previous studies have shown that many plant C2H2 ZFPs containing the EAR motif function as repressors, including *Arabidopsis* AZF1/2/3, ZAT7, and ZAT10/11/12.^45,49,50^ In contrast, IbZFP1 exhibits transcriptional activation activity and lacks EAR motif.^51^ Interestingly, rice ZFP245 acts as a transcriptional activator, despite the presence of EAR motif.^52^ A recent study of AtZAT14 showed that the function to promote ectopic cell death by transcriptional repression activation depends on both EAR motif and L-box.^53^ In our study, GmZFP1 and GmZFP3 contain EAR motif in the C terminal, while GmZFP2 and GmZFP4 do not, but the four GAL4DBD-C2H2 ZFP fusion proteins did not induce the 5×GAL4-LUC expression in *N. benthamiana* leaves, further research showed that all four C2H2 ZFPs possessed transcriptional repression activation. Amino acid sequence comparisons revealed three conserved α-helix regions exiting in GmZFP2, but whether these regions are associated with transcriptional repression function is still unknown. The results above imply that there may be other uncharacterized motifs besides EAR involved in transcriptional regulation.

The production of ROS is a key component of plant immunity, and the accumulation triggers programmed cell death (PCD), commonly known as the hypersensitive response (HR). In *N. benthamiana* leaves, overexpression of *GmZFP2* induced accumulation of H_2_O_2_ and expression of two HR marker genes *NbHSR203J* and *NbHIN1*, suggest a positive role in pathogen defense. C2H2 ZFPs have been found involved in ROS-scavenging responses to abiotic stresses such as salt, drought, and heat stresses.^18,54–56^ The results described above indicate the functional diversity of C2H2 ZFPs in response to biotic and abiotic stresses. RBOH is a crucial regulator of plant immune responses and serves as a primary catalytic enzyme responsible for the production of ROS. RBOH catalyzes the production of superoxide anions from oxygen, which are then converted to H_2_O_2_ in the apoplast catalyzed by superoxide dismutase.^57^ In this study, overexpression of *GmZFP2* resulted in the elevated expression of *NbRbohB*, which may suggest that *GmZFP2* can facilitate the production of ROS. We propose a hypothesis that *GmZFP2* may target unknown target proteins relying on transcriptional repression activation or direct protein interaction, thus relieving the inhibition of *RBOH*; alternatively, it may directly suppress the expression of key genes involved in ROS elimination, such as *APX*, leading to the accumulation of ROS, and further stimulating the plant immune response and augmenting resistance to infections. However, the mechanisms of *GmZFP2* in response to pathogens need to be further analyzed.

## Conclusion

In the present study, by using transcriptome data collected before and after inoculating two soybean varieties with different resistance levels to *P. sojae*, we analyzed the expression patterns of C2H2-ZFP family genes in response to *P. sojae* infection, and selected four candidate genes for further functional analysis. Among them, *Glyma.11G184000* (*GmZFP2*) displayed significant upregulation in both resistant and susceptible soybean varieties and possessed transcriptional repression activation. Transient expression in *N. benthamiana* leaves demonstrated that *GmZFP2* exhibits significant cell death- and ROS-inducing activities. Disease resistance evaluation results showed that *GmZFP2* significantly enhanced the resistance to *Phytophthora* pathogens in *N. benthamiana* leaves and soybean hairy roots. These results demonstrate *GmZFP2* plays an important role in plant resistance.

## Supplementary Material

Table S2.xlsx

Supporting information 1.pdf

Table S1.xlsx

## Data Availability

All the data are available in the article/Supplementary Materials.
