# Supplementary material for: Functional characterization of four soybean C2H2 zinc-finger genes in Phytophthora resistance
Source: Plant Signal Behav. 2025 Mar 20;20(1):2481185. doi: 10.1080/15592324.2025.2481185 (PMC11926910; doi:10.1080/15592324.2025.2481185)
Supplement: Supporting information 1.pdf [file KPSB_A_2481185_SM5035.pdf]

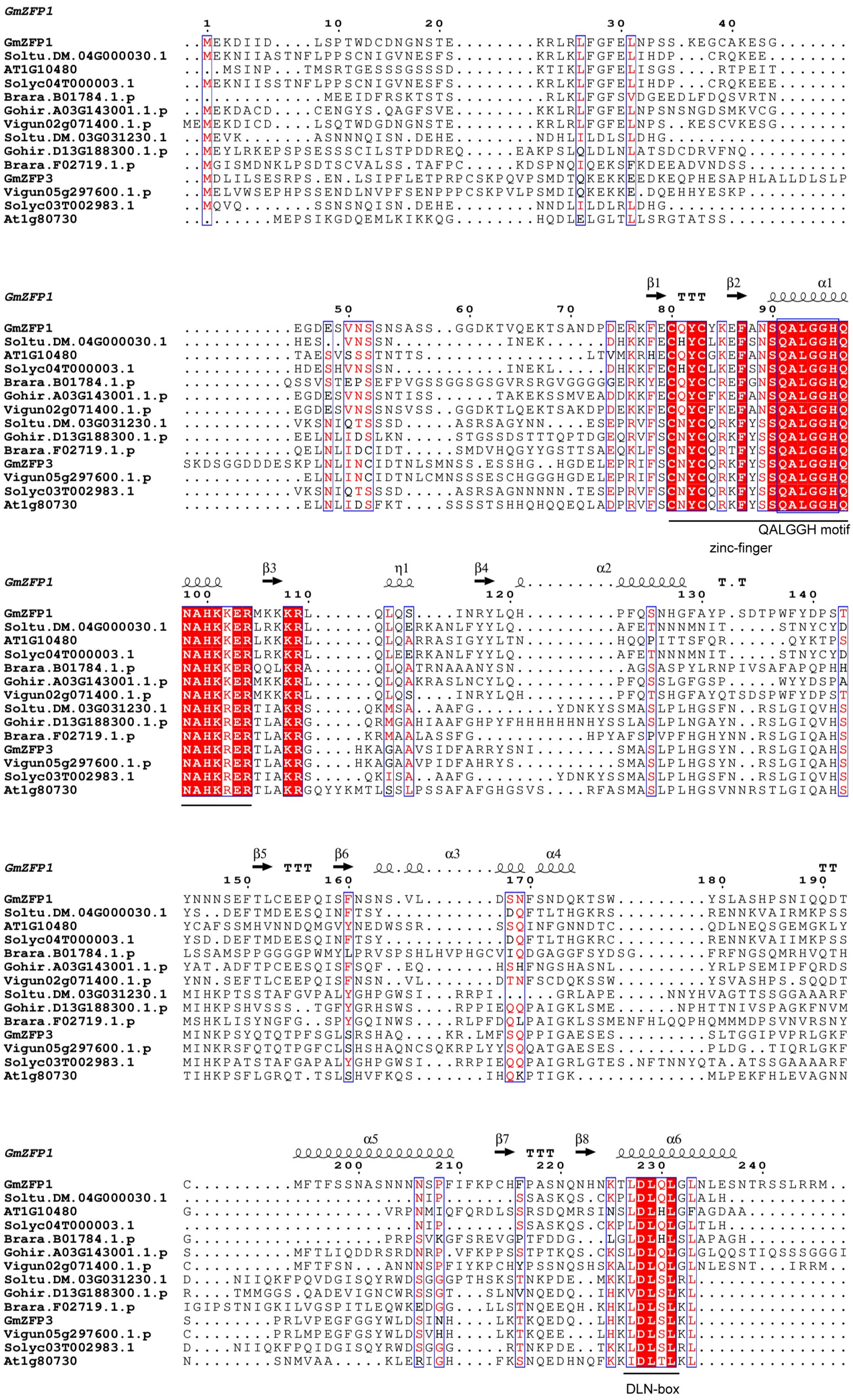
GmZFP1
GmZFP1
Soltu.DM.04G000030.1
AT1G10480
Solyc04T000003.1
Brara.B01784.1.p
Gohir.A03G143001.1.p
Vigun02g071400.1.p
Soltu.DM.03G031230.1
Gohir.D13G188300.1.p
Brara.F02719.1.p
GmZFP3
Vigun05g297600.1.p
Solyc03T002983.1
At1g80730
QALGGH motif
zinc-finger
DLN-box

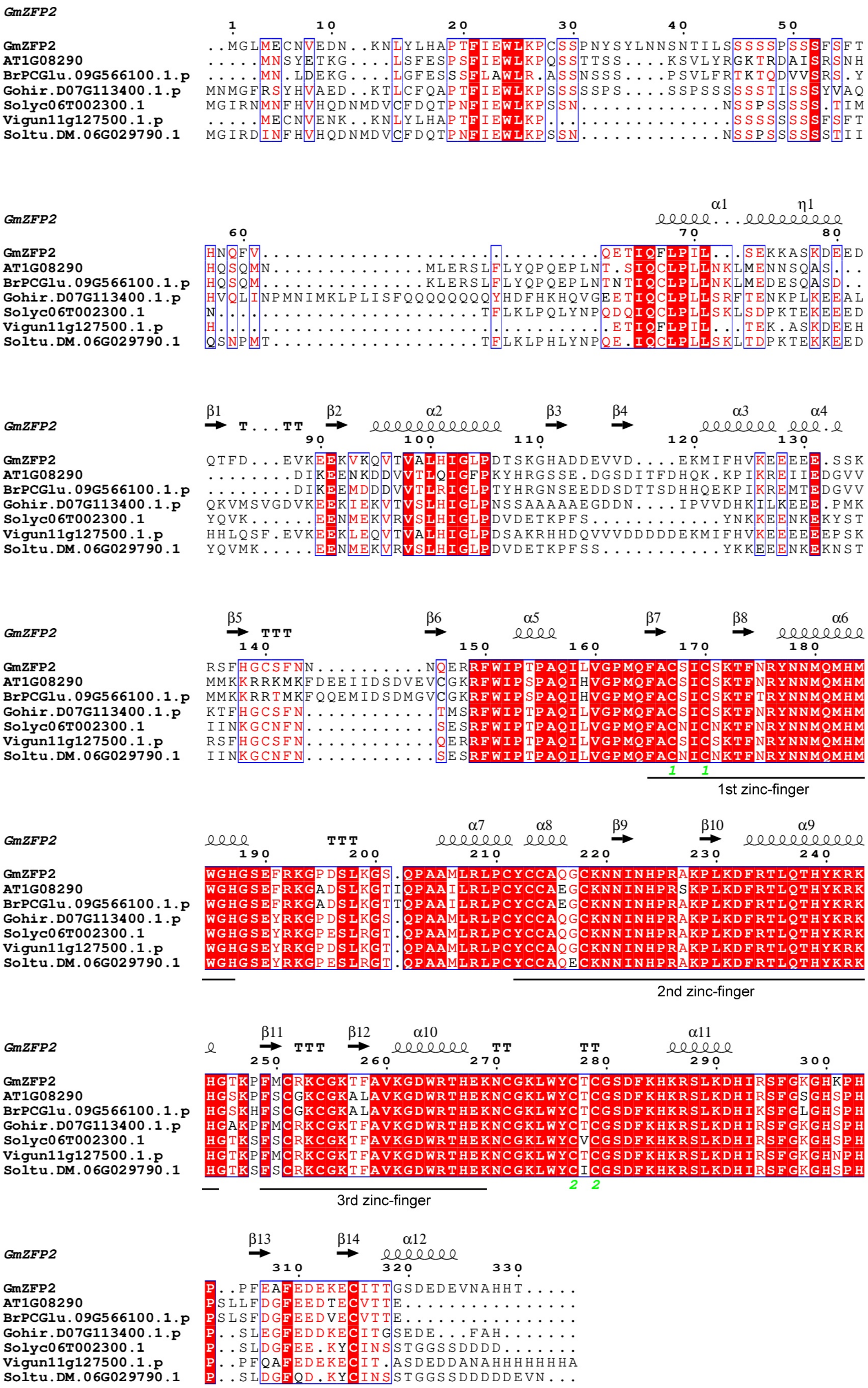

GmZFP2
1 10 20 30 40 50
GmZFP2 ..MGLMECNVEDN..KNLYLHAPTFIEWLKPCSSPNYSYLNNSNTILSSSSSPSSSFSFT
AT1G08290 .....MNSYETKG....LSFESPSFIEWLKPQSSTTSS....KSVLYRGKTRDAISRSNH
BrPCGlu.09G566100.1.p .....MN.LDEKG....LGFESSSFLAWLR.ASSNSSS....PSVLFRTKTQDVVSRS.Y
Gohir.D07G113400.1.p MNMGFRSYHVAED..KTLCFQAPTFIEWLKPSSSSSPS....SSPSSSSSSTISSSYVAQ
Solyc06T002300.1 MGIRNMNFHVHQDNMDVCFDQTPNFIEWLKPSSN.............NSSPSSSSS.TIM
Vigun11g127500.1.p .....MECNVENK..KNLYLHAPTFIEWLKP................SSSSSSSFSFT
Soltu.DM.06G029790.1 MGIRDINFHVHQDNMDVCFDQTPNFIEWLKPSSN.............NSSPSSSSSTII
α1 η1
60 70 80
GmZFP2 HNQFV...QETIQFLPIL...SEKKASKDEED
AT1G08290 HQSQMN...............MLERSLFLYQPQEPLNT.SIQCLPLLNKLMENNSQAS...
BrPCGlu.09G566100.1.p HQSQM...............KLERSLFLYQPQEPLNTNTIQCLPLLNKLMEDESQASD..
Gohir.D07G113400.1.p HVQLINPMNIMKLPLISFQQQQQQQYHDFHKHQVGEETIQCLPLLSRFTENKPLKEEAL
Solyc06T002300.1 N.......................TFLKLPQLYNPQDQIQCLPLLSKLSDPKTEKEEED
Vigun11g127500.1.p H...ETIQFLPIL...TEK.ASKDEEH
Soltu.DM.06G029790.1 QSNPMT..................TFLKLPHLYNPQE.IQCLPLLSKLTDPKTEKKEED
β1 T...TT β2 α2 β3 β4 α3 α4
90 100 110 120 130
GmZFP2 QTFD...EVKEEKVKQVTVALHIGLPDTSKGHADDEVVD....EKMIFHVKEEEEE.SSK
AT1G08290 ........DIKEENKDDVVTLQIGFPKYHRGSSE.DGSDITFDHQK.KPIKREIIEDGVV
BrPCGlu.09G566100.1.p ........DIKEEMDDDVVTLRIGLPTYHRGNSEEDDSDTTSDHHQEKPIKREMTEDGVV
Gohir.D07G113400.1.p QKVMSVGDVKEEKIEKVTVSLHIGLPNSSAAAAAEGDDN....IPVVDHKILKEEE.PMK
Solyc06T002300.1 YQVK......EENMEKVRVSLHIGLPDVDETKPFS...........YNKKEENKEKYST
Vigun11g127500.1.p HHLQSF.EVKEEKLEQVTVALHIGLPDSAKRHHDQVVVDDDDDEKMIFHVKEEEEEEPSK
Soltu.DM.06G029790.1 YQVMK.....EENMEKVRVSLHIGLPDVDETKPFSS...........YKKEEENKEKNST
β5 TTT β6 α5 β7 β8 α6
140 150 160 170 180
GmZFP2 RSFHGCSFNN..........NQERRFWIPTPAQILVGPMQFACSICSKTFNRYNNMQMHM
AT1G08290 MMKKRRKMKFDEEIIDSDVEVCGKRFWIPSPAQIHVGPMQFACSICSKTFNRYNNMQMHM
BrPCGlu.09G566100.1.p MMKKRRTMKFQQEMIDSDMGVCGKRFWIPSPAQIHVGPMQFACSICSKTFTRYNNMQMHM
Gohir.D07G113400.1.p KTFHGCSFN............TMSRFWIPTPAQILVGPMQFACSICSKTFNRYNNMQMHM
Solyc06T002300.1 IINKGCNFN............SESRFWIPTPAQILVGPMQFACNICNKTFNRYNNMQMHM
Vigun11g127500.1.p RSFHGCSFN............QERRFWIPTPAQILVGPMQFACSICSKTFNRYNNMQMHM
Soltu.DM.06G029790.1 IINKGCNFN............SESRFWIPTPAQILVGPMQFACNICNKTFNRYNNMQMHM
1 1
1st zinc-finger
TTT α7 α8 β9 β10 α9
190 200 210 220 230 240
GmZFP2 WGHGSEFRKGPDSLKGS.QPAAMLRLPCYCCAQGCKNNINHPRAKPLKDFRTLQTHYKRK
AT1G08290 WGHGSEFRKGADSLKGTIQPAAILRLPCYCCAEGCKNNINHPRSKPLKDFRTLQTHYKRK
BrPCGlu.09G566100.1.p WGHGSEFRKGADSLKGTTQPAAILRLPCYCCAEGCKNNINHPRAKPLKDFRTLQTHYKRK
Gohir.D07G113400.1.p WGHGSEYRKGPDSLKGS.QPAAMLRLPCYCCAQGCKNNINHPRAKPLKDFRTLQTHYKRK
Solyc06T002300.1 WGHGSEYRKGPESLRGT.QPAAMLRLPCYCCAQGCKNNINHPRAKPLKDFRTLQTHYKRK
Vigun11g127500.1.p WGHGSEYRKGPDSLKGT.QPAAMLRLPCYCCAQGCKNNINHPRAKPLKDFRTLQTHYKRK
Soltu.DM.06G029790.1 WGHGSEYRKGPESLRGT.QPAAMLRLPCYCCAQECKNNINHPRAKPLKDFRTLQTHYKRK
2nd zinc-finger
β11 TTT β12 α10 TT TT α11
250 260 270 280 290 300
GmZFP2 HGTKPFMCRKCGKTFAVKGDWRTHEKNCGKLWYCTCGSDFKHKRSLKDHIRSFGKGHKPH
AT1G08290 HGSKPFSCGKCGKALAVKGDWRTHEKNCGKLWYCTCGSDFKHKRSLKDHIRSFGSGHSPH
BrPCGlu.09G566100.1.p HGSKHFSCGKCGKALAVKGDWRTHEKNCGKLWYCTCGSDFKHKRSLKDHIKSFGLGHSPH
Gohir.D07G113400.1.p HGAKPFMCRKCGKTFAVKGDWRTHEKNCGKLWYCTCGSDFKHKRSLKDHIRSFGKGHSPH
Solyc06T002300.1 HGTKSFSCRKCGKTFAVKGDWRTHEKNCGKLWYCVCGSDFKHKRSLKDHIRSFGKGHSPH
Vigun11g127500.1.p HGTKPFMCRKCGKTFAVKGDWRTHEKNCGKLWYCTCGSDFKHKRSLKDHIRSFGKGHNPH
Soltu.DM.06G029790.1 HGTKSFSCRKCGKTFAVKGDWRTHEKNCGKLWYCICGSDFKHKRSLKDHIRSFGKGHSPH
2 2
3rd zinc-finger
β13 β14 α12
310 320 330
GmZFP2 P..PFEAFEDEKECITTGSDEDEVNAHHT.....
AT1G08290 PSLLFDGFEEDTECVTTE...............
BrPCGlu.09G566100.1.p PSLSFDGFEEDVECVTTE...............
Gohir.D07G113400.1.p P..SLEGFEDDKECITGSEDE...FAH.......
Solyc06T002300.1 P..SLDGFEE.KYCINSSTGGSSDDDD.......
Vigun11g127500.1.p P..PFQAFEDEKECIT.ASDEDDANAHHHHHHA
Soltu.DM.06G029790.1 P..SLDGFQD.KYCINSSTGGSSDDDDDEVN...

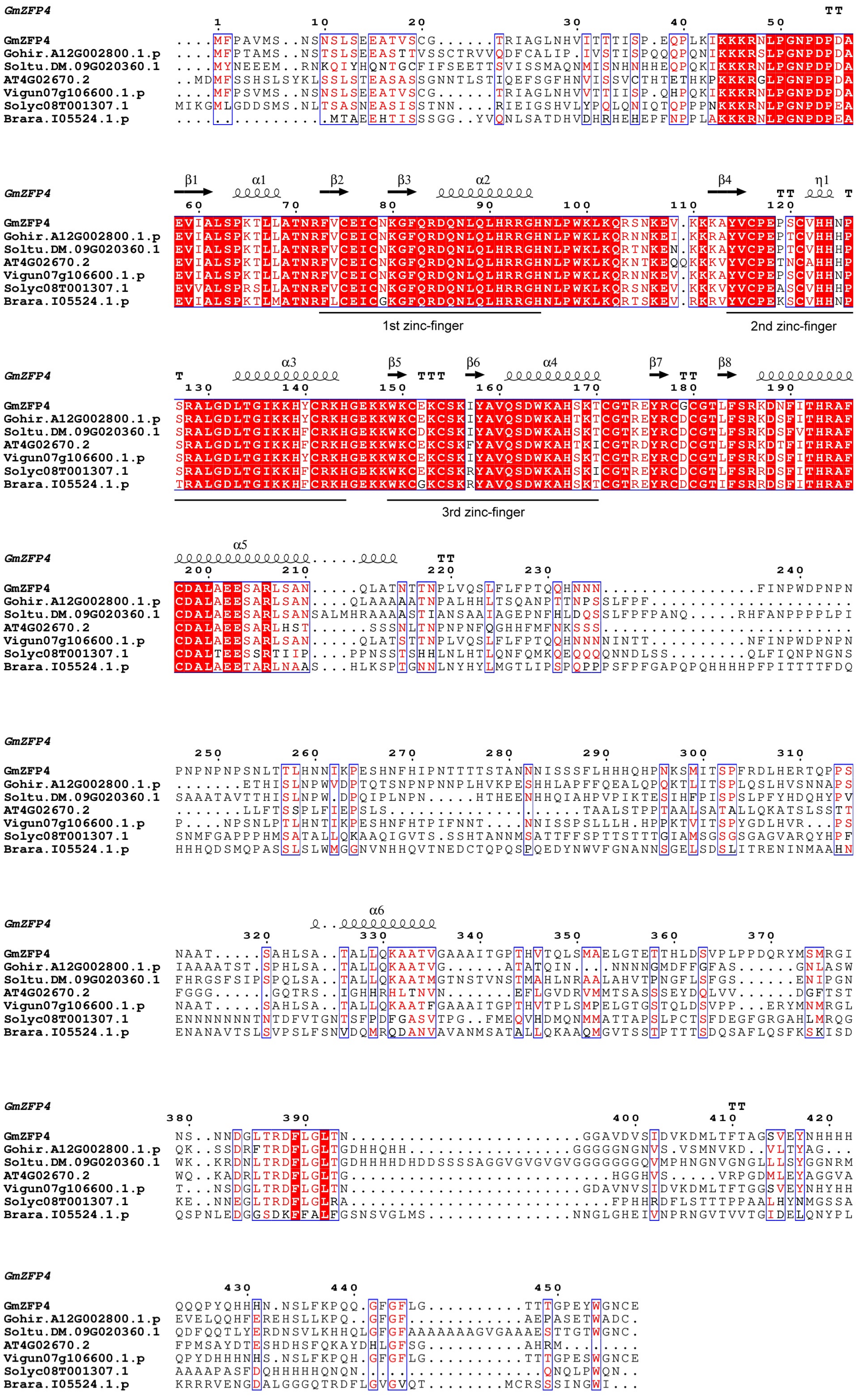
GmZFP4
GmZFP4
Gohir.A12G002800.1.p
Soltu.DM.09G020360.1
AT4G02670.2
Vigun07g106600.1.p
Solyc08T001307.1
Brara.I05524.1.p
1st zinc-finger
2nd zinc-finger
3rd zinc-finger
